# Embryo futures and stem cell research: the management of informed uncertainty

**DOI:** 10.1111/j.1467-9566.2011.01367.x

**Published:** 2012-01

**Authors:** Kathryn Ehrich, Clare Williams, Bobbie Farsides, Rosamund Scott

**Affiliations:** 1 King's National Institute for Health Research Patient Safety and Service Quality Research Centre, King's CollegeLondon; 2 Department of Sociology and Communications, Brunel UniversityLondon; 3 Brighton and Sussex Medical SchoolLondon; 4 Centre for Medical Law and Ethics, King's CollegeLondon

**Keywords:** embryo donation, futures, human embryonic stem cell research, restricted consent, informed uncertainty

## Abstract

In the social worlds of assisted conception and stem cell science, uncertainties proliferate and particular framings of the future may be highly strategic. In this article we explore meanings and articulations of the future using data from our study of ethical and social issues implicated by the donation of embryos to human embryonic stem cell research in three linked assisted conception units and stem cell laboratories in the UK. Framings of the future in this field inform the professional management of uncertainty and we explore some of the tensions this involves in practice. The bifurcation of choices for donating embryos into accepting informed uncertainty or not donating at all was identified through the research process of interviews and ethics discussion groups. Professional staff accounts in this study contained moral orientations that valued ideas such as engendering patient trust by offering full information, the sense of collective ownership of the National Heath Service and publicly funded science and ideas for how donors might be able to give restricted consent as a third option.

## Introduction

The discourse around human embryonic stem cell (hESC) research is characterised by narratives of hope and hype (Braude *et al.* 2005, Prainsack *et al.* 2008) and the futurity of such narratives, most often in terms of their scientific, political and commercial ‘promise’ and ‘potential’ (Geesink *et al.* 2008). For example, plans for long-term UK investment in hESC research emphasise its promise for future utility in healing and cure (Department of Health 2005). Yet Michael *et al.* (2007) argue that stem cell scientists themselves have articulated vague and opaque futures which perform uncertainty and wariness in the present. Similarly, Waldby and Mitchell (2006) highlight uncertain futures or destinations as a key characteristic of hESC research: ‘In the case of embryo donation for stem cell research, the embryo is simply a starting point for an expandable network of cell lines whose destination is unknowable’ (2006: 71).

We have considered elsewhere the discourse on embryos in this professional field in terms of multiple framings (Goffman 1974) of embryos as social objects (Mead 1934), arguing that the constitution of embryos as moral work objects is emergent and contextually contingent (Ehrich *et al.* 2008). Embryos may be framed within personal and local world-views but also in relation to the bigger frame of an official account of the embryo that accepts and legitimates certain practices (Ehrich *et al.* 2008). For example, Jasanoff (2005) argues that ‘in a neat display of co-production’ (2005: 152), acceptance in the UK of the destruction of ‘spare’ embryos and the 14-day limit for embryo research was achieved through framing the pre-14-day-old embryo as a ‘pre-embryo’. By co-production Jasanoff (2005, 2006) is referring to the concept of forms of social order and technology or science being generated and enacted in mutually constituting ways. Embryos are viewed as ‘spare’ clinically and at the same time broader policies and social practices confirm the legitimacy of asking women and couples to donate spare embryos for hESC research. It might be inferred from this situation that since clinical practice, laboratory science and regulations are all in place to support embryo donation for hESC research, then the ethical implications have been or could be resolved and would be consistent throughout the location of the legislation's jurisdiction. However, our research participants have alerted us to remaining tensions in clinical practice, particularly in relation to uncertainties about the futures for embryos donated to hESC research, the hESCs derived from them and how these can be dealt with in ethically acceptable ways in their contemporary work with potential embryo donors.

Embryos can be framed in terms of the meanings of different futures being accomplished, contested, performed or avoided (Kitzinger and Williams 2005, Williams 2005). In her foreword to a landmark collection on the performative character of future expectations of science (Brown *et al.* 2000), Adam urges social scientists to engage with questions about the unknowable futures created by techno-scientific innovation. She suggests that, in the interests of a socially responsible future, we need to attend to, render visible and explain taken-for-granted social processes of the present, by asking:

… how the future is created, constructed, contested, colonised and consumed; how it is materialised, managed and ‘mastered’; how opportunities are created for some at the expense of others; how uncertainties, indeterminacies and contingencies are handled. (Adam 2000: xii)

Articulations about the future can thus be used to illuminate social relations and policy in the present and may also be important in shaping future policy and practice. In the social worlds of assisted conception and stem cell science uncertainties proliferate and particular framings of the future may be highly strategic. In this article we explore meanings and articulations of the future using data from our study of ethical and social issues implicated by the donation of embryos to hESC research in three linked assisted conception units (ACU) and stem cell laboratories in the UK. We focus on the contexts and world-views that professional staff in assisted conception and hESC research draw upon when contemplating the futures for donated embryos and hESC lines and advising potential donors of spare embryos to hESC research. Our aim is to show how, in this case, framings of the future by professionals in this field inform their management of uncertainty and to explore some of the tensions this involves in practice.

Social and ethical issues for donors or potential donors of embryos for research have been addressed in the literature, for example, religious and cultural aspects (Barratt *et al.* 2004); ethical and policy issues (Cohen 2009, McLeod and Baylis 2007); attitudes and decision-making (de Lacey 2006, 2007a, 2007b) and informed consent (Haimes and Taylor 2009, Heng 2006). However, this article contributes to a better understanding of how professional staff negotiate social and ethical aspects of their work in a field characterised by uncertainty and controversy as well as excitement and hope. It adds to a small but growing body of work investigating clinical and scientific professionals’ experiences and beliefs about social and ethical aspects of their work in the closely related fields of reproductive technologies, hESC research and genetics (Ehrich *et al.* 2006, 2007, 2008, 2010a, 2010b, Franklin and Roberts 2006, Kerr and Franklin 2006, Roberts and Throsby 2008, Stephens *et al.* 2008, Williams *et al.* 2008).

## Clinical context

In the UK, the Human Fertilisation and Embryology Authority (HFEA) oversees the regulation of all licensed infertility, or assisted conception, clinics. Clinics that arrange for embryos not used for a particular woman's or couple's fertility treatment to be donated for research must ensure that informed consent is given and documented. Fulfilment of the requirements to obtain informed consent from embryo donors to have their spare embryos used for research may be the responsibility of nurses, clinicians or stem cell coordinators depending on local arrangements. Before treatment commences an initial consent process is undertaken using a standard form issued by the HFEA. If patients indicate willingness to consider donating embryos to research in general they may be approached later in a further process of consent to a specific research project (for further details about the consent process see Franklin *et al.* 2008, Ehrich *et al.* 2011).

The embryos used for hESC research are those that women or couples cannot, or choose not to, use for their own treatment (for instance, because they cannot afford to freeze embryos after their embryo transfer or because the clinic has a ‘minimum number’ freezing policy or because their embryos have been tested for genetic conditions through pre-implantation genetic diagnosis, or PGD). The assessment of their likely viability is determined by embryologists and clinicians in the ACU. Embryos that are judged to be unsuitable either for transfer to the woman's womb at 3–6 days after fertilisation or for cryopreservation for a later ‘frozen embryo transfer’ may be discarded or donated to research, including hESC research (for further details of this assessment process see Cutting *et al.* 2008, Ehrich *et al.* 2007, 2010b, Svendsen and Koch 2008).

## Methods

This article addresses one aspect of a multidisciplinary ethnographic study that explored the views, values and practices of professional staff in relation to embryo donation for research purposes, particularly for hESC research. The study sites were three ACUs in teaching hospitals in England, which offer a mixture of National Health Service (NHS), privately, or ‘self-funded’ NHS treatment, and three stem cell laboratories at the universities associated with these hospitals. The clinics provide a range of assisted conception services including *in vitro* fertilisation (IVF). Following national and local research ethics committee approvals, the study methods included clinic and laboratory observations, interviews and ethics discussion groups (EDGs) (Alderson *et al.* 2002) with staff from ACUs and linked stem cell laboratories in the UK. Staff disciplines included nursing, obstetrics and gynaecology, embryology, genetics, stem cell science, counselling and clinical and research management.

As a multidisciplinary team comprised of social scientists, ethicists, clinical specialists and a legal expert, our aim was to explore the social processes, meanings and institutions that frame and produce the consideration of ethical problems in these settings. It must be acknowledged that our approach, as a multidisciplinary team of researchers, was implicated in the framing and production of our data in terms of ethical issues. However the framing of issues in this way arose as a result of the mutual constitution of the meaning of our data with our study participants. The process began with identifying what staff themselves defined as ethical issues, the exploration of these in interviews and in EDGs. In this way the issues we explored were not simply imposed from outside the research setting but arose within that context.

The participants were recruited by group introductions to the study, followed by individual approaches from the main researcher. Observations in the clinics and laboratories were carried out to gain knowledge of everyday practices and scientific background and to identify the issues that were explored further in the interviews and EDGs. The interviews were conducted by the main researcher as guided conversations (Lofland and Lofland 1984), lasting between 1 and 2 hours. An informal interview guide was used to ask participants open-ended questions on topics such as what they thought of as ethical issues in their work, their views on embryo donation for research and hESC research in particular; any personal views or religious beliefs that were pertinent to this issue in their work and whether they foresaw future ethical, legal, social, technological or clinical issues for hESC research and its possible applications. The researcher drew on her experience of research using social science methods to ask follow-up questions exploring the world-view of each participant, and how their values, views and experiences informed their daily work. The interviews were recorded and transcribed and the transcripts were analysed for content to produce themes for further discussion in the EDGs, especially topics suggested by staff.

The EDGs differed from the interviews in that the facilitator (BF, a philosopher in our study team with long experience of facilitating similar groups addressing ethical issues in healthcare settings) followed up contributions with probes informed more by her philosophical orientation. The EDGs are not focus groups but are based on the approach of a philosophy seminar where participants are asked to consider issues such as consistency, coherence and the value they attach to different concepts and choices. The study methods therefore combine eliciting participants’ views in interviews with a sociologist/anthropologist with the exploration and development of the topics from a different disciplinary direction with their colleagues. This allowed each group to reflect on their views and practice and to learn more about those of their colleagues. Both the researcher and the EDG facilitator presented themselves as independent researchers with no ethical or policy stance on the issues but with a strong interest in how staff themselves view the ethical dilemmas that arise in their work. The rationale for EDG recruitment was to ensure that, as far as possible, each group included staff from a mixture of disciplines, which was found to be appreciated by staff in previous projects (Alderson *et al.* 2002, Ehrich and Williams 2010). The EDGs lasted for two hours and participants were asked to keep the proceedings confidential.

For our analysis of the interview and EDG transcripts we used thematic analysis aspects of the framework approach (Ritchie and Spencer 1994) following close readings of the interview and EDG transcripts. The themes reported here emerged from this analysis as the study proceeded and were discussed in our team meetings. While we do not claim that the quotes we present here are statistically representative of the views of staff in our sites or in the UK generally, they illustrate common themes that arose from the fieldwork at these sites. All quotes are from interviews except where indicated by referring first to the participant's occupation and study number and then to the EDG number.

We note that for this article we use the terms ‘women and couples’, ‘embryo donors’ and ‘patients’ to refer to people receiving treatment from the ACU and PGD service; and ‘staff’ or ‘participants’ to describe the members of staff who engaged in the interviews and EDGs. We acknowledge that some readers might prefer other conventions. We also emphasise that some differences in clinical practice between sites may be an effect of the timing of our fieldwork. Finally, we note that embryos can be donated for a range of research projects, such as to improve assisted conception techniques, but we focus in this article on hESC research.

## Findings

We interviewed 44 members of staff and held six EDGs at our study sites between February 2008 and October 2009. The total number of participants in the EDGs was 25, including six members of staff who were not interviewed before taking part in an EDG. The groups ranged in size from three to six participants and each participant attended only one group. All staff groups were represented in the EDGs as a whole, and in most groups a good mixture of disciplines was achieved.

The study participants were engaged (in the practical sense) to varying degrees with embryo donation to hESC research. Although a limited number of staff were directly involved in the formal consenting process for donation of embryos to hESC research, this necessarily takes place in contexts in which a broader range of staff talk with women and couples and give information to potential donors about possible futures for their embryos, and in which there is a high degree of awareness of the ethical and sometimes controversial aspects of their work. The issues we address here were therefore pertinent to all of our participants.

Nurses in two ACUs and clinicians in the third ACU reported from here (at the times when our fieldwork took place) were responsible for discussing the initial HFEA consent forms. In two clinics donations for hESC research were not being sought at the time we commenced our interviews although they had been previously. It was envisaged in these two clinics that research staff (that is, scientists and research managers from the hESC research laboratory) would be responsible for providing further information and obtaining separate consent for specific research projects. Near the end of our interviewing phase at one clinic a stem cell coordinator was appointed and the hESC research donations resumed. At the third clinic a stem cell coordinator had been in place before our study commenced and was responsible for obtaining consent from patients for embryo donations to hESC research.

Our analysis draws on the approach introduced earlier, that is, to open up spaces for consideration of some of the social and ethical aspects of work in this field. The findings are organised into themes grouped together into two main sections: an exploration of the future uncertainties voiced by study participants, followed by a section on how staff manage forms of uncertainty.

## Future uncertainties

The uncertainty that has long been recognised as a feature of much medical knowledge and medical work (Fox 1959), including assisted conception, is compounded when considering IVF and hESC research because of the long-term future implications of both fields. These twin dimensions of uncertainty and futurity in IVF and hESC research connect profoundly at the point where potential donors are asked if their embryos may leave the ‘pregnancy trail’ (Cussins 1996) to join the ‘research trail’ (Parry 2006). At the ‘IVF-stem cell interface’ (Franklin 2006) the professional staff whose work entails advising women and couples contemplating donation are faced with numerous dilemmas. We address some key issues from this study regarding uncertainty at this interface elsewhere, including anticipating how donors might be informed about incidental research findings (Ehrich *et al.* 2010a) and social ethical and scientific dimensions in the contingent classification of ‘spare’ embryos (2010b). We focus in this first section on some uncertainties about hESC research in comparison to other research in ACUs; doubts about the long-term effectiveness and reliability of regulation in this field and some of the values and moral orientations staff voiced that led to questions about resources and benefits in hESC research.

## Long-term uncertainties in hESC research compared to other research

All three sites in our study were in linked universities and teaching hospitals so that research was a familiar feature of their environment. However, most of our study participants regarded hESC research as a particularly uncertain field compared to other kinds of research. Most research in ACUs relates to the immediate treatment goals of the units, that is, improving fertility treatments, and might contribute in the medium term to new treatments that could help their own patients, so staff had little difficulty with patients being asked to take part in studies. By contrast, hESC research was seen as potentially helpful, but to a broader range of health problems and only in the long term.

A key temporal issue relating specifically to hESC research concerns long-term uncertainties as a consequence of the goal of the infinite perpetuation of the cell lines. The open-ended nature of hESC research was compared by ACU staff to more familiar kinds of research in which research on embryos must be completed within 14 days:

It's very different to any kind of research that we have approached patients for in the past where we've been keeping the embryos in culture for maybe an extra 2 or 3 days, but then still allowing them to perish. (Embryologist 4)

Stem cell scientist 35 compared the futurity of donated gametes to another couple's treatment with the implications of hESC research and the finality of PGD research:

I can see that the problem of donating to another couple is that you may have a sibling or half sibling with gametes out there in the world, that your children don't know about or you might not know about … you have a part, a genetically related person continuing on in time, which is more comparable to deriving a stem cell line, because then you also have your genes and your cells growing somewhere forever … whereas if you're talking about … PGD research, embryos are used and destroyed … and you get your end point that's your information, it's finished. I would imagine that's far less problematic because you don't have anything out there, with time. (Stem cell scientist 35, EDG 5)

The long-term uncertainties of hESC presented staff with unique challenges in communicating with donors about the future implications:

I think with other projects … say, if you're looking at biochemistry inside the embryo and then it's discarded or allowed to perish, whereas with stem cell research, although you're actually removing a part of the embryo … so the embryo itself isn't viable at that point, but the actual patient's cells will be growing, forever growing and developing. I think that's quite a difficult issue to explain to patients, and for them to come to terms with as well … So I think there's a lot more ethical issues surrounding stem cell research than there are with other embryo research projects. (Embryologist 2)

With PGD, embryo screening, the embryo gets destroyed after it's used in the research, but with stem cells, because they're going to become immortal … we may make stem cells, okay that's fine, but then what are they going to be used for? And that answer we can't give them. We can in the short term, but in the long run they could be used for anything. (Research manager 29, EDG 3)

These comments illustrate the relative lack of established narratives available to staff about the kinds of uncertainty associated with hESC research. This raised issues that were different from other research they had approached patients for in the past and were difficult to explain. The participants were more comfortable discussing donation for research where they felt they could offer women and couples a more definite set of possibilities. Some staff comparing the futurity aspect of hESC research to other kinds of research used the metaphor of closure, emphasising the importance for patients of knowing the limits of their commitment. The futurity of hESC research offers less closure than other kinds of research and some staff compared this aspect to the long-term uncertainties associated with donating embryos for other people's fertility treatment; for example, the thought that a person genetically related to embryo donors could exist without their having contact or knowledge of them.

### Regulation and future uncertainties

Accounts of how staff view future uncertainty in relation to future implications of embryo donation indicated their concern about the long-term reliability of regulatory safeguards. Counsellor 6 compared the example of possible future implications of sperm donation and changes in regulation regarding donor anonymity with futurity issues in hESC science:

There could be new regulations introduced; the law is constantly being revised, society's attitudes have changed, technology develops, nobody can give you a 100% guarantee that what you're consenting to today will remain … Our knowledge is limited … even if you ring-fence it, we can't guarantee that there won't be some future piece of legislation that decrees that all existing stem cell lines are to be used and they're going to make it retrospective because it's deemed that this is going to be so, of such benefit to society. (Counsellor 6)

In this account, a key implication of the futurity of sperm donation and stem cell science, by comparison, was the risk of future changes in legislation. Future political processes could not be relied upon to honour the current values embedded in the current legislation and her use of the metaphor of ring-fencing echoes the concept of closure. Her concern illustrates the difficulty for staff who would prefer to be able to help patients make choices about the future on a more certain basis and who recognise that closure, in the sense of being able to protect decisions from the effects of future legislation, is ultimately impossible. Many of our participants regarded the current UK model of regulation of science and fertility treatment as providing a framework of safety:

I believe in the British standard, I do … infertility, the licensing laws, I think are done quite wisely in this country. I can't say so much for Europe, but I think the British, the British regulations are good; I trust them. (Counsellor 28, EDG 3)

However, as this comment illustrates, concerns about hESCs deposited in the UK Stem Cell Bank and then being used by scientists in other countries with different regulations were voiced by several participants. Embryologist 7 raised this topic in EDG 1:

Who is going to go to the lab in [names country] and check what research they're doing there if they've bought a line in? Because they haven't got an HFEA. (Embryologist 7, EDG 1)

Stem cell scientist 14 then responded to Embryologist 7:

There's nothing to stop those people doing research on it that they haven't proposed. So the UK Stem Cell Bank would be able to say, ‘Okay you can use it for that project’. But once you've got that cell line in your lab, it proliferates and proliferates and proliferates, and you could get somebody who comes in and goes, ‘Oh I fancy doing those experiments’, and would just do them if they weren't regulated. [Embryologist 7]'s right. And there's no way that we could regulate that in this country. (Stem cell scientist 14, EDG 1)

The global dimension of hESC research thus raised further tensions because containment could not be guaranteed outside of the boundary of UK regulation, illustrating one aspect of our study participants’ awareness of the political and social embeddedness of hESC research (including differences between the UK and other countries).

### Resources and benefits of hESC science

Some ambivalence was voiced about who would benefit from hESC science. One feature of this concerned commercial aspects of hESC science, for example, regarding payment to women not undergoing IVF for eggs to be used for research:

I can't really imagine any circumstances where somebody would want to do that purely for advances in research … if money then starts to come into it, then … suddenly it becomes like a commodity and so I think I would be very uncomfortable with that idea. (Embryologist 34, EDG 6)

Widening this out to the potential benefits of hESC science for treatments, participants considered who would benefit beyond financial gain:

It's not just about money, it's about who you want to help. I think it's easier for us to see a purpose in helping a specific couple than nameless people who we've never met, however big that number would be. And also what you're using them for is not just money, it's who it's for and the use of them as well. (Nurse 48, EDG 6)

Clinician 8 addressed these questions drawing on a set of beliefs about the relationship of science to the NHS and public funding. He argued that since public money and public effort had gone into hESC research some of the commercial gains should benefit the NHS. He went on to develop his views drawing on a much broader concept of the social contract that he believes is under threat in the UK:

I think it's very disappointing that we've not been able to exploit modern medical science without essentially patients or taxpayers having to pay way over the odds for the profit, for the benefits, because of this modern move to make everything commercial. It's a trend in society which is all pervasive. It's not only in medicine … I think some people in society actually deserve a free lunch and it's part of being civilised that we give it to them … you know, our hospital's broke, every hospital in Britain is broke, there's always need for more money. (Clinician 8)

In this account, the NHS is publicly owned and constituted for the benefit of UK citizens and stands more broadly as a metaphor for the social contract between the public and government. This resonates with Hoeyer and Lyno's (2006) work on blood and other tissue donation to the Swedish Medical Biobank, in which they emphasise the importance of understanding donation in the particular context of the Swedish welfare state. Clinician 8's belief in the ideal of reciprocity and his expression of values informing an idea of common ownership of the NHS informs his problematic stance to the support of science, and thus he explicitly raises the political embeddedness of the production of hESC science.

The discussion of futurity issues in the interviews and EDGs allowed values and moral orientations to be explored in a context that freed staff from concerns about overwhelming or burdening patients with too much uncertainty. It allowed staff to demonstrate their commitment to both personal and collective values but also their ability to keep issues that are unresolved under debate and avoid complacency. It also elicited accounts about future uncertainties in hESC research that were contextualised beyond the more limited issues concerned with consent procedures in the clinic and instead to comment from a broader context informing their ethical views that included kinship and friendship relations and commitment to the NHS.

## The management of future uncertainty

We suggested in the previous section that staff had a relative lack of established narratives to draw upon when communicating with potential embryo donors about the particular long-term uncertainties involved in hESC research. Participants’ concerns about the reliability of regulation in protecting patients’ long-term interests and questions about who stands to benefit from this research also inform the way in which the staff in this context manage these uncertainties. In this section we explore the management of uncertainty, including the use of more established repertoires for managing other forms of uncertainty in this field; the importance of openness and trust and invoking caution to manage the problem of ‘informed uncertainty’– a concept that has been used in relation to consent processes in biobanking and participation in clinical trials – in embryo donation consent processes.

### Clinical management of uncertainty

Fertility treatment and PGD entail a great deal of management of uncertainty and participants in this study gave a number of accounts of how this is achieved. For example, nurses, clinicians and embryologists described how they built into consultations information to lower patients’ expectations about the number of embryos that would be likely to succeed:

It's a lot of education for a start about that … saying that the embryos are observed from day to day and … saying that unless their embryos get to that eight cell stage, then they don't really offer them such a good chance of getting pregnant … you've got to get all that information into them. And so that lowers, in their mind, the potential of each individual embryo … if you were going to talk about research first of all, they think they don't want research because they want every embryo because every embryo is a baby … you have to lower their expectations there as well and … get them to think realistically about their embryos. (Nurse 1)

The staff aim to achieve awareness in patients of substantial uncertainty of success and thus some detachment of hope from individual embryos. Nurse 1 makes the link between lowering expectations for each embryo with a willingness to think about research. Once women and couples start to ‘think realistically about their embryos’ the nature of staff requests to consider donating some embryos that cannot be used for their treatment changes. As Nurse 1 makes clear, the introduction of uncertainty and detachment of hope as part of the management of expectations can be drawn upon when staff ask women and couples to donate these embryos to research, because staff anticipate that the patients will have learned not to think of each one of them as a ‘baby’.

The management of expectations through ‘education’ about the uncertainty of individual embryos creating a pregnancy is an important part of the ongoing communication with patients in relation to taking them through the treatment pathway:

If you don't explain it to them, they assume that you freeze all the embryos. And if they have a negative test, you don't want them to come back and say, ‘Oh but we thought we'd got them all frozen’, and you say, ‘Well no actually, none of them were suitable, we donated them to research’, because then it looks like you're covering up things. So I think you have to be very careful how you explain it to them … That's quite good here that the embryologists have daily contact with the patient. And I always go to egg recovery and explain that every day there will be a drop off in the number that are suitable. (Embryologist 7)

In the face of inherent uncertainties in the treatment, staff are concerned to manage patient expectations by bringing as much clarity to the communication process as possible. This resonates with Franklin and Roberts’ (2006) finding in their study of PGD that patients often seemed to value clear acknowledgement of uncertainty and that this could enhance their trust in clinical staff. ‘Education’ and clarity of communication are used in the management of expectations and building trust. The achievement of lowered expectations and trust in the treatment process underpins the ethical basis upon which staff can then communicate with women and couples about uncertain future implications of donating embryos to research.

### Openness and trust

We have seen that openness and trust feature in the clinical management of uncertainty, as in the quote above when Embryologist 7 expressed her concern that women and couples truly understand the basis on which their embryos are donated for research. Participants in this study voiced the unanimous hope that patients would trust them to prioritise the treatment goal of achieving pregnancy over research. Embryologist 50 opened up this sensitive issue of trust with her colleagues in EDG 6:

If they truly trusted us and truly knew that we were out for all good intentions for them, then [patients] would be more inclined to sign for research … I think maybe they think, ‘Well what if they don't use it? I'm sure they do things, you know, behind closed doors. Look at what happened in Japan, when they had that hybrid, the human and the monkey. Do you think they're going to do that with our embryos?’ (Embryologist 50, EDG 6)

The values of openness and trust could lead to tensions between the wish to be fully informative and the thought that patients could be frightened if they were informed of future possibilities:

I think it's important for the patient to be aware that these things could happen, but to express it to the patient in a way that you don't put them off donating embryos to research [laughs] because you could frighten them to death. (Embryologist 2)

Another source of possible tension stemmed from staff members wishing to be open but holding personal views that they felt should not interfere with their professional roles. Some staff commented on how they managed such tensions, for example, expressing their personal views and possible objections to particular applications of stem cell science while conforming to a professional stance in which one would not express personal views when speaking with patients. In the context of the EDGs, such comments could be interpreted as framing personal views within the bigger frame of collegiate discussions or alternatively within what could be understood as the semi-public context of the EDGs and possibly being quoted in publications.

### Informed uncertainty and invoking caution

Within their accounts of uncertainties and futurity in hESC science, versions of the future emerged in which boundaries were placed around some aspects of the future that can be known or guaranteed and some that cannot. One of the responses to the problem that this raises was to make sure that women and couples were fully informed about what cannot be known about how hESCs derived from their embryos might be used, including the uncertainties about how future legislation might affect the use of donated embryos as discussed above.

Another response, cited by several participants, including nurses, embryologists and clinicians, was to split potential donors’ options into two frames: either give consent to ‘spare’ embryos being used for hESC research, accepting informed uncertainty, or refuse to donate at all. Embryologist 4's comments illustrate this polarised framing of options:

[Patients] need to understand that they haven't got any legal right over any developments or money that's to be made from therapies … I think they have to be informed on every single one of those issues, and if they're not completely happy with it, then they don't do it … But then I think as long as they know that and understand that, that's fine … we should look to the future as much as we can … at some point we will lose control of the cells that have come out from their embryos. And if they feel uncomfortable with that, then they shouldn't do it. (Embryologist 4)

In this formulation, the onus is on the professional staff to inform women and couples about their legal rights, commercial and ethical issues, and the lack of control over future uses of cells, but once they are so informed it is then open to potential donors to refuse on the grounds of avoiding potential (and uncertain) future harms. This ‘better safe than sorry’ approach, which we would express as invoking caution, chimes with some of the less extreme elements of the precautionary principle (Andorno 2004, Saunders 2000). The precautionary principle has been defined in a number of ways (Manson 2002) but most often it is seen as underpinning safeguards against threats of harm to human health or the environment from scientific or technological developments not fully proved to be safe (Science and Environmental Health Network 1998). This tendency in regulation has been conceptualised as a balance between risk and opportunity (Stirling 2007) and criticised for potentially stifling innovation (Holm and Harris 1999).

In this case, the staff involved in seeking consent for embryos to be donated to research cannot claim to have proof that no harm to patients will result from their donation and some staff had concerns about the possible consequences that may not become apparent for many years. Invoking caution (in a less extreme form than the use of the precautionary principle in regulation) can thus be used as a resource by offering a pragmatic way of dealing with the consequences in the present of the messiness of the uncertain future, as well as avoiding the possibility of blame should harm occur in the future (Tubiana 2000).

Some participants described how they emphasised possible safeguards in communication with patients, such as regulatory containment, while acknowledging but downplaying uncertainties. For example, Embryologist 7 described how staff can present ‘a standard answer’ to potential donors that emphasises the safety provided by the HFEA and the Act, even though she had her doubts about how much could be assured outside of the political boundary of the UK. The recognition of these tensions led some participants to consider whether there could be instances when embryo donors to hESC research should be offered choices beyond what might be termed the ‘all or nothing’ acceptance of informed uncertainty or ‘better safe than sorry’ rejection of donation, allowing them to specify or prohibit particular uses of stem cells derived from their embryos. For example, the UK Human Fertilisation and Embryology Act (as amended in 2008) prohibits the use of gametes derived *in vitro* from hESCs for reproductive treatment, but several participants in our study questioned whether embryo donors could be sure they would never be used for treatment in the future and thought patients would be particularly concerned about this issue:

I think they would have great problems to think that their stem cells were being, their genetic material is making sperm for other people. (Clinician 15, EDG 1)

The significance for patients of distinguishing between other types of tissue and reproductive tissues raised the possibility that considering more options for consent regarding very specific issues such as gamete production might allow both staff and patients more room for manoeuvre (see also Ehrich *et al.* 2011). Allowing specific forms of restricted consent would be consistent with staff values that emphasise openness, informed choice and respect for potential embryo donors’ interests in their own treatment over any research goals. We acknowledge that a less polarised resolution might seem more difficult to operate, because awareness of uncertainty can lead to a response of restricting choices to make consenting processes more manageable. However, the acknowledgement of uncertainty can also be the basis of a more relational model of autonomy and consent (Ehrich *et al.* 2007, Franklin and Roberts 2006) that pays attention to the wider social contexts in which reproductive technologies both shape and are affected by such choices (Mackenzie and Stoljar 2000, Sherwin 1998). Our data suggest that elements of this are already a feature of professional practice in this field.

## Discussion and conclusion

In contrast to the background in hESC research of unspecifiable promise, uncertainty and future orientation, a more pragmatic approach is employed by staff whose work involves discussing with women and couples the fate of embryos that are thought least likely to succeed in creating a pregnancy and are therefore possible candidates for donation to hESC science. Participants in this research illustrated some of the tensions between wishing to provide full information for women and couples and building trust by focusing on the treatment goals of assisted conception and PGD and downplaying future uncertainties in hESC science. Alongside this pragmatic, day-to-day framing, more subtle tensions between uncertain futures, fully informed consent and the management of uncertainty were evident in our discussions with staff at these three sites. In parallel to the framing of their discussions with patients about the future according to more boundaried scenarios, staff held personally and socially constituted concerns and uncertainties about embryo and hESC futures in which a number of personal, local and broader social or cultural resources came into consideration.

Invoking caution as a means of managing informed uncertainty about the future was identified through the research process of interviews and discussion groups. Of interest here is that instead of the more formal mechanism of adopting the precautionary principle through legislation in order to restrain the activities of scientists, which some would argue threatens to stifle innovation (Holm and Harris 1999, Tubiana 2000), the staff invite patients to use less formal but nevertheless rather polarised decision-making formulations themselves. This enables staff to conform to the duty in clinical practice to act, without having to shoulder the burden of proof that no harm can come to patients as a consequence of donating their embryos to hESC research (ter Meulen 2005).

Embryos can be framed in terms of the meanings of different futures being accomplished, contested, performed or avoided. Jasanoff (2005) points out that the various alternative ways in which human embryos may be framed accommodates the interpretive flexibility in which policies are formed. As a further example of co-production we have seen in this article how the staff prioritise the framing of embryos as treatment objects over their potential as research objects, which helps to create a category of ethically acceptable spare embryos. Inviting patients to exercise caution also allows staff to emphasise informed uncertainty, which may help to build trust. The staff demonstrated awareness of sensitivities concerning the political and social embeddedness of embryos and the hESCs they may produce, for example, when they framed embryos as commercial and therapeutic objects in distant arenas to their origins in fertility treatment, or when framing their comments on hESC research in the context of their commitment to the NHS. This could be seen as illustrating a key attribute of ‘Mode 2’ knowledge production summarised by Jasanoff (2005) as an awareness in participants of science of:

… the social implications and assumptions of their work … just as publics have grown more conscious of the ways in which science and technology affect their interests and values. (2005: 232)

Participants in this study expressed moral orientations that valued ideas such as engendering patient trust by offering full information, the sense of collective ownership of the NHS and publicly funded science and ideas for how donors might be able to give restricted consent. They also expressed concerns about possible future uses of the technology and choices available to women and couples regarding the future use of hESCs derived from their embryos, for example, the implications of fertility treatment using gametes derived *in vitro* from hESCs; and raised questions about the resources needed for hESC science and who might ultimately benefit from it. In some ways the futures represented are characterised by a lack of control, in contrast to the containment of uncertainties in the present, for example, by lowering expectations of success in fertility treatment. Managing the choices made by potential donors in terms of invoking caution, nevertheless, leaves some staff with unresolved questions. We see the opening up of these social and ethical concerns by staff as an articulation of the uncertainties and ambivalence that characterise this field. We would argue that some staff are working in a way that is consistent with a more relational model of autonomy and consent that also enhances their ability to manage uncertainty in this context. We argue that this relational approach may be seen as a vital part of the contemporary co-production (Jasanoff 2005, 2006) of science and policy.
